# Improving the Communication of Dental Findings in Pediatric Dentistry by Using Intraoral Scans as a Visual Aid: A Randomized Clinical Trial

**DOI:** 10.3390/dj12010015

**Published:** 2024-01-17

**Authors:** Nelly Schulz-Weidner, Marina Gruber, Eva May Schraml, Bernd Wöstmann, Norbert Krämer, Maximiliane Amelie Schlenz

**Affiliations:** 1 Dental Clinic—Department of Pediatric Dentistry, Justus Liebig University, Schlangenzahl 14, 35392 Giessen, Germany; norbert.kraemer@dentist.med.uni-giessen.de; 2 Dental Clinic—Department of Prosthodontics, Justus Liebig University, Schlangenzahl 14, 35392 Giessen, Germany; marina.gruber@dentist.med.uni-giessen.de (M.G.); bernd.woestmann@dentist.med.uni-giessen.de (B.W.); 3 Department of Oral and Maxillofacial Surgery, Justus Liebig University, Klinikstrasse 33, 35392 Giessen, Germany; eva.schraml@uniklinikum-giessen.de

**Keywords:** intraoral scanners, dental caries, molar incisor hypoplasia, clinical study, digital dentistry, pediatric dentistry, oral health, interdisciplinary study, caries diagnostics, healthcare research

## Abstract

The aim of this first randomized clinical trial (RCT) was to determine whether intraoral scans (IOS) can be used as a visual aid to improve the communication of dental findings in pediatric dentistry. Therefore, 60 children (mean age 10.1 ± 3.3 years) and their accompanying parents/primary caregivers (PGs) were examined between July 2022 and February 2023. Patients were randomly allocated to two groups: half of the participants were informed of the children’s dental findings including treatment plans by verbal explanation alone (control group, *n* = 30), while the other half were informed using IOS (Trios 4, 3Shape) as a visual aid to support the verbal explanation (study group, *n* = 30). Both groups then completed a questionnaire regarding their children’s diagnosis, treatment needs, planned therapy, and oral hygiene. Statistical analysis was performed using a *t*-test (*p* < 0.05). Overall, there was a significant difference between the two groups (*p* < 0.001) in terms of understanding the current oral situation of their children. While 85.5 ± 17.3% (mean ± standard deviation) of the answers were correct in the study group, only 57.2 ± 17.8% of the participants in the control group were capable of answering the questions correctly. In particular, the control group had difficulties answering the questions about treatment needs and therapy correctly. Within the limitations of this first pilot study, IOS can be clearly recommended as a visual aid to improve the communication of dental findings with PGs in pediatric dentistry.

## 1. Introduction

The impact of visual aids in health communication for adults was highlighted in a recent systematic review and meta-analysis. For example, images improved knowledge, understanding, and number of recall visits in patients with chronic diseases [1]. Well-informed patients are more likely to be engaged in the management of their health conditions, make more informed and better decisions, and ultimately contribute to a higher quality of care. Furthermore, literature suggests that a lack of understanding of the need for treatment, particularly among younger or more anxious patients, may lead to skepticism and lower acceptance [1]. In addition to the benefits of knowledge transfer and the clarification of complex issues, there is a risk of this being used to guide or manipulate the patient. In addition, in most countries it is a legal requirement that patients must be fully informed in order to be able to exercise their right to self-determination.

The merits of traditional visual aids have long seen them recognized as useful adjuncts to written information, especially for people with low literacy skills and in contexts where visualization is critical [2]. For example, the benefit of visual aids in improving patient understanding and education in the context of surgical procedures and chronic disease management has already been demonstrated in clinical trials [3,4].

Although providing information about dental findings is a routine part of every dentist’s practice, medical language is often difficult for patients to understand. In addition, language barriers sometimes pose additional problems. Unlike adult dentistry, pediatric dentistry involves children as patients and their parents/primary caregivers (PGs), which is sometimes even more challenging. PGs play a critical role in the management of children’s oral health care, especially for dental treatment procedures. Therefore, effective communication is the cornerstone of quality dental care and oral health outcomes. In pediatric dentistry, children’s oral health literacy is inherently limited. Therefore, the involvement of PGs in information seeking and the delivery of oral health messages from dentists to children is important. Building on these modalities, new technologies offer additional opportunities for improved means of visual communication [5].

However, the use of dental study models, which is common in dentistry, is often too abstract because patients cannot see their own oral situation. Live intraoral video camera images can help here, but they only display a small area of the mouth. Furthermore, some patients may feel uncomfortable seeing real images of their mouth on a screen [6]. Therefore, new technologies such as intraoral scanners could help as a visual aid to improve communication between patients and dentists by providing a visual aid that goes beyond traditional approaches. Capturing color 3D virtual models of the entire jaw, with additional features such as caries diagnostic tools, is more reminiscent of a computer game than a real oral situation.

The use of intraoral scanners for more than just taking digital impressions is a growing field. For example, visualization of orthodontic or prosthetic treatment options through smile design tools has already been implemented in many intraoral scanner software. Clinical smile design studies have shown an improvement in comprehension when using intraoral scanners as a visual aid [2,7,8,9,10,11].

In addition to visualization for better comprehension, visual aids also play an important role in diagnosis. In the case of caries management, some intraoral scanners offer caries diagnostic tools for the detection of occlusal carious lesions integrated into the hardware and software of the intraoral scanner [12,13], which could help to detect and monitor enamel carious lesions at an early stage to allow minimally invasive treatment options [14,15,16,17,18,19].

Besides caries, pediatric dentistry faces another challenging diagnostic situation. Molar incisor hypomineralization (MIH) is a therapeutic challenge and requires proper diagnosis. Careful diagnostic differentiation should be performed before initiating any dental treatment [20,21]. For a better grading, Steffen et al. [22] standardized the diagnostic criteria and treatment needs for MIH and developed a classification system that links lesion severity to the *Treatment Need Index* (TNI). However, this MIH-TNI is based on visual examination [21]. Therefore, an informed treatment decision can be made using the therapy scheme of Bekes et al. [22], which takes into account both the clinical findings of the extent of defects and hypersensitivity, as well as increased caries risk.

A good and reliable diagnosis is essential because the young age and possible diseases in pediatric dentistry require special empathy with the children and PGs. It has been shown time and again that parental consent to necessary treatment is strongly dependent on a clear explanation. According to a study conducted by Wang et al. [23], patients who were only informed by verbal explanation had more negative behaviors than those who received additional pictorial information.

Thus, visualization appears to improve patient comprehension in the medical setting, and it is only a matter of finding an appropriate, feasible tool for daily patient care in the pediatric dentistry.

The objective of this initial randomized clinical trial (RCT) was to determine if intraoral scans (IOS) could be used as such a visual aid to improve the communication of dental findings in pediatric dentistry. It was hypothesized that the use of IOS would improve communication and thus increase the PGs’ comprehension of the children’s dental findings and treatment plan.

## 2. Materials and Methods

The present study was conducted according to the CONSORT 2010 statement guidelines; Supplementary Table S1 presents a completed checklist.

### 2.1. Study Design

The RCT was conducted in a parallel, single-center design between July 2022 and February 2023. Our study was registered in the German Register of Clinical Trials (DRKS00029333) and the local ethics committee of JLU (Ref. No. 46/20) approved the study. Inclusion criteria for subjects were an age of 5–18 years, attendance at their regular dental check-ups at the Department of Pediatric Dentistry, Justus Liebig University Giessen (JLU, Giessen, Germany), and that they and their accompanying PGs were informed in advance about the study procedure and asked to participate voluntarily. Written informed consent was obtained if the response was positive. Exclusion criteria were a lack of consent to participate and being younger than five or older than 18 years.

**Figure 1:**
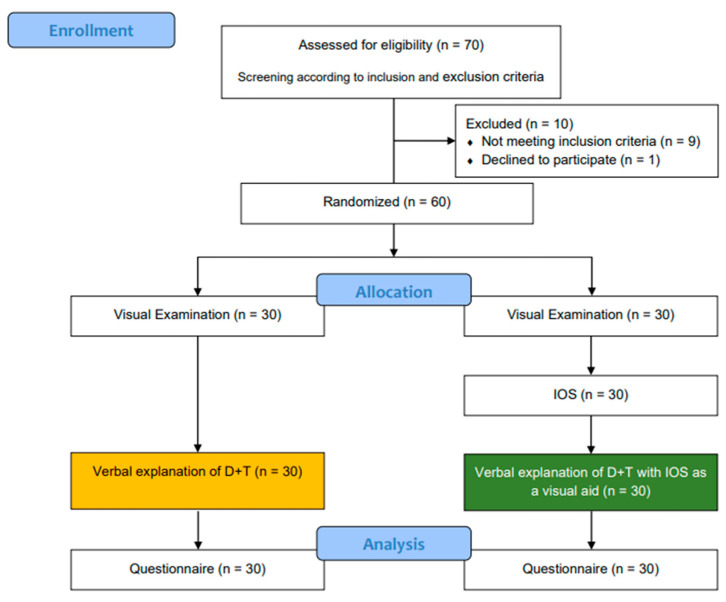
Flow diagram of the investigation (IOS = intraoral scans, D + T = dental findings and treatment plan) according to CONSORT 2010 statement.

Each group of patients received a different explanation of the dental findings and the planned therapy. They were randomly assigned to the control group (verbal explanation) or the study group (verbal explanation with IOS). Figure 1 shows the study procedure.

All appropriate therapeutic options were then enumerated and offered, regardless of the method of explanation used.

### 2.2. Visual Examination

The established visual examination was performed under standardized conditions with standardized illumination (25,000 lx) using a dental mirror and an air syringe. Caries experience was determined using the decayed-, missing-, filled-teeth (DMF-T/dmf-t) index showing caries lesions, working based on either untreated (number of decayed teeth) or treated (filled teeth or missing teeth extracted as a result of caries) teeth [24]. In addition, structural anomalies such as MIH or oral findings due to orthodontic or surgical issues were noted [25].

Prior to this study, the two dentists (M.G., N.S.-W.) who performed the examination were calibrated according to an established diagnostic procedure (visual examination for caries detection and visual examination for MIH examination). In this way, intra-rater reliability (consistent findings by each examiner) and inter-rater reliability (consistent diagnoses by different examiners) could be determined [26]. The intensity of agreement was thus almost perfect, according to Landis and Koch [27] (Cohen–Kappa coefficient (κ) > 0.81).

All dental findings and planned therapy were recorded on a control questionnaire for later comparison with the PG questionnaire.

### 2.3. Intraoral Scanning

After visual examination by one examiner (N.S.-W.), all children were scanned using a trained examiner (M.G.). Prior to scanning, the IOS Trios 4 (version 20.1.4, 3Shape, Copenhagen, Denmark) was color-calibrated. Scanning was performed, starting with the occlusal surfaces of the jaw, followed by the oral surfaces, and ending with the buccal surfaces.

### 2.4. Explanation of Dental Findings

All children and PGs were informed about the dental findings and the planned therapy according to a predefined scheme. The findings were first discussed in the same order of upper right, upper left, lower left, and lower right, moving quadrant by quadrant, and the therapy recommendation was given analogously. After enrollment, the children were randomly assigned to one of the two groups. Although each group received the same explanation of the dental findings and planned therapy, in the control group children and PGs received only a verbal explanation, whereas in the study group IOS were used in addition to the verbal explanation.

### 2.5. Questionnaire

Directly after the explanation of dental findings and planned therapy, PGs of both groups were asked to complete a paper-based questionnaire. This questionnaire comprised six questions regarding their children’s diagnosis, treatment needs, planned therapy, and oral hygiene (see Supplementary material). While the first two questions required the PGs to mark the correct answers on an illustration of the upper and lower jaw, the remaining questions provided answer choices. Three different versions of the questionnaires were designed, with different illustrations depending on the children’s dentition (primary, mixed, or permanent dentition). 

Subsequently, the PGs’ questionnaires were evaluated in terms of which and how many questions they answered correctly according to the control questionnaire (see Section 2.2).

### 2.6. Statistical Analysis

IBM SPSS Statistics (version 29, IBM, Armonk, NY, USA) was used for statistical analysis and a *t*-test (*p* < 0.05) was performed. Data are presented as absolute and relative frequencies. Mean and standard deviation were also calculated.

## 3. Results

60 children (mean age 10.1 ± 3.3 years) examined between July 2022 and February 2023 were included in this RCT. They already presented 441 permanent teeth and 276 teeth in the primary dentition stage, which were visually inspected for caries, MIH and other oral findings. 

**Figure 2:**
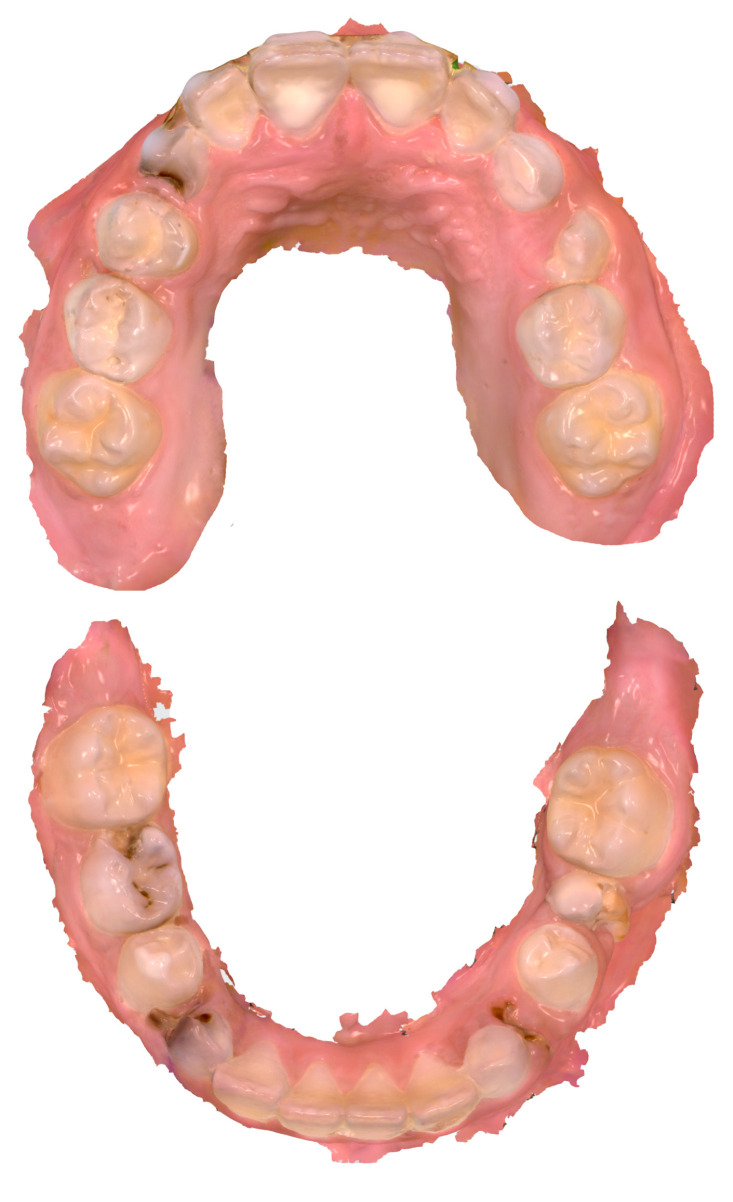
Example of intraoral scans (Trios 4) as a visual aid in an 8-year-old child with multiple carious lesions.

In the primary dentition, 114 teeth were decayed, 13 were missing due to caries and 24 were filled. Of the permanent teeth, 42 showed carious lesions, 3 were missing, and 35 were filled. MIH was present in 28 teeth. Additionally, there were two cases of lateral occlusion. Figure 2 shows an example of IOS as a visual aid in an 8-year-old child with multiple carious lesions.

**Figure 3:**
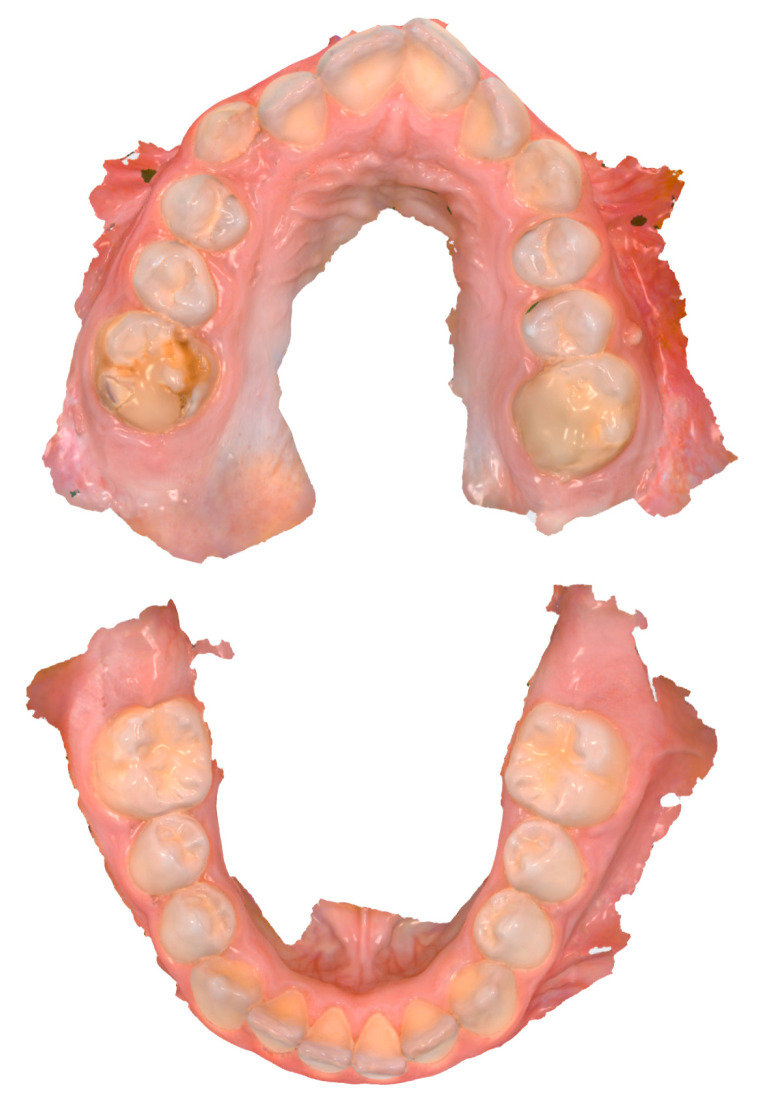
Example of intraoral scans (Trios 4) as a visual aid in an 11-year-old child presenting MIH in regions #16 and #26 (FDI scheme).

To image MIH lesions in IOS, Figure 3 displays a clinical example of an 11-year-old child.

**Figure 4:**
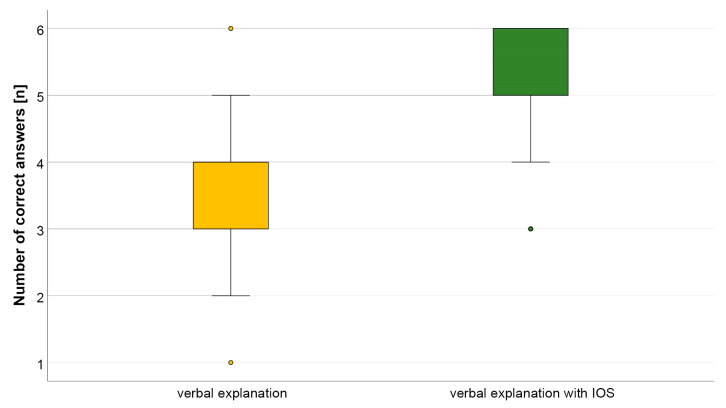
Boxplot diagram of overall data of correct answers of questionnaire distributed to control group (verbal explanation) and study group (verbal explanation with IOS).

A significant difference between the control group (verbal explanation) and the study group (verbal explanation with IOS) could be detected regarding the number of correct answers in the questionnaire (Figure 4). While 85.5 ± 17.3% (mean ± standard deviation) of the answers were correct in the study group, only 57.2 ± 17.8% of the participants in the control group could answer the questions correctly.

**Figure 5:**
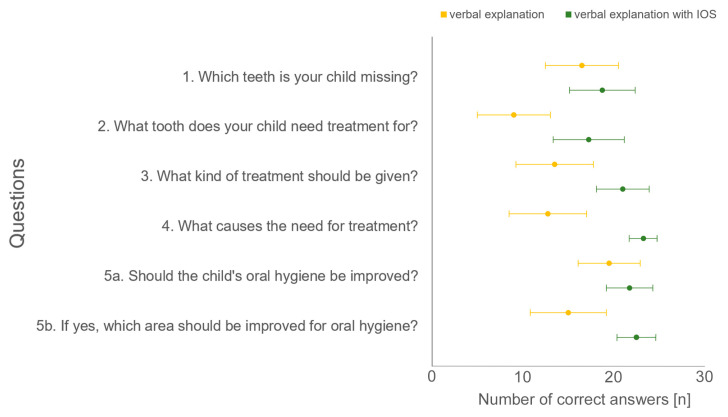
Number of correct answers (maximum *n* = 30) for control group (verbal explanation) and study group (verbal explanation with IOS) distributed to the six questions asked.

In particular, the control group had difficulties answering the treatment needs and therapy questions (question number 2–4) correctly (Figure 5).

## 4. Discussion

Studies have shown that the use of visual aids such as pictures or models can improve understanding. Although talking about dental findings is a daily routine for dentists, it is often difficult for patients to understand their oral situation [9,10,11]. The results of the present study illustrate that IOS are useful tools for visualizing dental findings in pediatric dentistry and improve the understanding of diagnosis and treatment planning for PGs. This seems to be particularly important with regard to the significance of parental understanding, especially in vulnerable patient groups and patients with language barriers. Visual aids could improve the communication of treatment risks to people with limited language skills and medical knowledge [28]. 

The benefits of visual communication are already being used in many ways in dentistry and recorded images and models are effective tools for diagnosis, treatment planning, and communication. For example, in the field of esthetic dentistry, it has been shown that visualization has a positive effect on the understanding of diagnosis and therapy. Digital smile design, a software-supported procedure, can be used to digitally show patients potential esthetic optimizations of their tooth positions. This allows the patient to visualize the final treatment outcome in advance. Coachman et al. [10] describe this procedure as a useful tool for improving patient education and motivation, and thus for improving communication with patients in general. Sousa Dias and Tsingene [11], as well as Jafri et al. [2], also describe increased patient understanding and acceptance of treatment as a result of this procedure.

In this study, a significantly higher understanding of the children’s oral situation was shown by the group of PGs who were informed about their child’s diagnosis and treatment planning with visual aids using IOS. This group showed better results in all of the questions than the group of PGs who received only verbal information without visual aids. Visualization tools seemed to be particularly useful, when the information was given in detail such as which tooth was affected or what areas should be improved with regard to oral hygiene. The support of visualization also significantly improved understanding regarding the pending treatment. Also, the conditions that caused the planned treatment were understood better, which may result in better treatment acceptance. IOS of individual patients are suitable for presenting dental conditions with greater specificity and in a fully personalized manner. Multiple studies have shown that IOS were found to be comparable to visual clinical examination of the oral situation of school children [29]. However, IOS for documentation purposes alone are not yet routinely performed in clinical, public health, or research settings, and can be particularly challenging in very young children. 

In addition, the use of IOS requires not only that it is available, but also that the user knows how to use it. However, as many practices are prosthetic and/or orthodontic, there will be colleagues familiar with IOS. In this study, the use of the scanner required hardly any additional time, as the examiner routinely used the scanner on a daily basis. Therefore, we assume that our model can facilitate patient–provider and practitioner–provider interactions because it is easily accessible to all parties. We believe it has significant potential to improve the overall quality of dental care. Moreover, in terms of vulnerable children, visualization could help to improve their understanding of concepts such as ‘risk factors’ and ‘being at risk’ and they have particular difficulty grasping the complexity of health risks [30].

Not only PGs have expressed interest in receiving information via digital technologies in the past [31]. It was also shown that children display greater acceptance of medical treatment when using digital technologies [32]. Moreover, patients whose dental treatment included IOS reported higher comfort and less chairside time compared to conventional impression techniques [33]. Especially when dealing with younger or more anxious patients, a skeptical attitude towards treatment may lead to a lower acceptance of treatment due to a lack of understanding of the need for treatment [2]. Special conditions such as MIH call for intensive long-term treatment. If the affected teeth are visualized to the patient and their caregivers during previous examinations, this may lead to an improved acceptance of the planned treatment and thus to better preservation of the teeth.

Thus, the study’s outcome also supports the fact that teledentistry has begun to emerge and may play a key role in maintaining oral health in pediatric dentistry [34]. Especially when access to face-to-face care is limited due to location or social and language boundaries, teledentistry as part of established screening programs may improve oral health in the long term.

In the present study, the DMF-T/dmf-t index was used for visual examination. Therefore, assessing severity and caries activity could not be determined, which must be seen as a limitation of the present study. Additionally, a further limitation was that only the PGs and not the children were interviewed. Therefore, future studies should develop a specific questionnaire for children. Furthermore, image quality was not considered to have an effect on the results.

Finally, multicenter studies including other areas of dentistry should be conducted following this to investigate a larger number of participants and to avoid bias. Long-term studies investigating actual treatment acceptance after visualized informed consent are needed. Further research should reflect children’s comprehension and take into account other factors such as language barriers.

## 5. Conclusions

Within the limitations of this RCT, IOS can clearly be recommended as a visual aid to improve the communication of dental findings with PGs in pediatric dentistry as it significantly increases comprehension, especially regarding treatment issues.

## Data Availability

The datasets of this article are available from the corresponding author on a reasonable request.

## Supplementary Materials

The following supporting information can be downloaded at: https://www.mdpi.com/article/10.3390/dj12010015/s1, Completed CONSORT 2010 statement guideline checklist Table S1.

## References

[1] Hibbard J.H. (2003). Engaging health care consumers to improve the quality of care. Med. Care.

[2] Jafri Z., Ahmad N., Sawai M., Sultan N., Bhardwaj A. (2020). Digital Smile Design-An innovative tool in aesthetic dentistry. J. Oral. Biol. Craniofac. Res..

[3] Bernhard J.C., Isotani S., Matsugasumi T., Duddalwar V., Hung A.J., Suer E., Baco E., Satkunasivam R., Djaladat H., Metcalfe C. (2016). Personalized 3D printed model of kidney and tumor anatomy: A useful tool for patient education. World J. Urol..

[4] Glaser J., Nouri S., Fernandez A., Sudore R.L., Schillinger D., Klein-Fedyshin M., Schenker Y. (2020). Interventions to Improve Patient Comprehension in Informed Consent for Medical and Surgical Procedures: An Updated Systematic Review. Med. Decis. Making.

[5] Horowitz A.M., Kleinman D.V. (2008). Oral health literacy: The new imperative to better oral health. Dent. Clin. N. Am..

[6] Rozier R.G., Slade G.D., Zeldin L.P., Wang H. (2005). Parents’ satisfaction with preventive dental care for young children provided by nondental primary care providers. Pediatr. Dent..

[7] Leisenberg D., Groß D. (2017). Visualisierungen und Visualisierungsstrategien in der Zahnheilkunde. Ethik Medizin.

[8] Shorey R., Moore K.E. (2009). Clinical digital photography today: Integral to efficient dental communications. J. Calif. Dent. Assoc..

[9] Clark J.M., Paivio A. (1991). Dual coding theory and education. Educ. Psychol. Rev..

[10] Coachman C., Calamita M., Ricci A. (2018). Digital Smile Design: A Digital Tool for Esthetic Evaluation, Team Communication, and Patient Management. Ronald E. Goldstein’s Esthetics in Dentistry.

[11] Sousa Dias N., Tsingene F. (2011). SAEF—Smile’s Aesthetic Evaluation form: A useful tool to improve communications between clinicians and patients during multidisciplinary treatment. Eur. J. Esthet. Dent..

[12] Schlenz M.A., Schupp B., Schmidt A., Wöstmann B., Baresel I., Krämer N., Schulz-Weidner N. (2022). New Caries Diagnostic Tools in Intraoral Scanners: A Comparative In Vitro Study to Established Methods in Permanent and Primary Teeth. Sensors.

[13] Michou S., Lambach M.S., Ntovas P., Benetti A.R., Bakhshandeh A., Rahiotis C., Ekstrand K.R., Vannahme C. (2022). Author Correction: Automated caries detection in vivo using a 3D intraoral scanner. Sci. Rep..

[14] Suese K. (2020). Progress in digital dentistry: The practical use of intraoral scanners. Dent. Mater. J..

[15] Gimenez T., Piovesan C., Braga M.M., Raggio D.P., Deery C., Ricketts D.N., Ekstrand K.R., Mendes F.M. (2015). Visual Inspection for Caries Detection: A Systematic Review and Meta-analysis. J. Dent. Res..

[16] Innes N.P.T., Chu C.H., Fontana M., Lo E.C.M., Thomson W.M., Uribe S., Heiland M., Jepsen S., Schwendicke F. (2019). A Century of Change towards Prevention and Minimal Intervention in Cariology. J. Dent. Res..

[17] Pitts N. (2009). Detection, Assessment, Diagnosis and Monitoring of Caries.

[18] Kuhnisch J., Ekstrand K.R., Pretty I., Twetman S., van Loveren C., Gizani S., Spyridonos Loizidou M. (2016). Best clinical practice guidance for management of early caries lesions in children and young adults: An EAPD policy document. Eur. Arch. Paediatr. Dent..

[19] Schwendicke F., Splieth C., Breschi L., Banerjee A., Fontana M., Paris S., Burrow M.F., Crombie F., Page L.F., Gatón-Hernández P. (2019). When to intervene in the caries process? An expert Delphi consensus statement. Clin. Oral. Investig..

[20] Steffen R., Krämer N., Bekes K. (2017). The Würzburg MIH concept: The MIH treatment need index (MIH TNI): A new index to assess and plan treatment in patients with molar incisior hypomineralisation (MIH). Eur. Arch. Paediatr. Dent..

[21] Dulla J.A., Meyer-Lueckel H. (2021). Molar-incisor hypomineralisation: Narrative review on etiology, epidemiology, diagnostics and treatment decision. Swiss Dent. J..

[22] Bekes K., Steffen R., Krämer N. (2023). Update of the molar incisor hypomineralization: Würzburg concept. Eur. Arch. Paediatr. Dent..

[23] Wang S.J., Briskie D., Hu J.C., Majewski R., Inglehart M.R. (2010). Illustrated information for parent education: Parent and patient responses. Pediatr. Dent..

[24] WHO Oral Health Surveys: Basic Methods. https://www.who.int/publication/i/item/9789241548649.

[25] Lygidakis N.A., Wong F., Jälevik B., Vierrou A.M., Alaluusua S., Espelid I. (2010). Best Clinical Practice Guidance for clinicians dealing with children presenting with Molar-Incisor-Hypomineralisation (MIH): An EAPD Policy Document. Eur. Arch. Paediatr. Dent..

[26] Schaefer G., Pitchika V., Litzenburger F., Hickel R., Kühnisch J. (2018). Evaluation of occlusal caries detection and assessment by visual inspection, digital bitewing radiography and near-infrared light transillumination. Clin. Oral. Investig..

[27] Landis J.R., Koch G.G. (1977). The measurement of observer agreement for categorical data. Biometrics.

[28] James X., Hawkins A., Rowel R. (2007). An Assessment of the Cultural Appropriateness of Emergency Preparedness Communication for Low Income Minorities. J. Homel. Secur. Emerg. Manag..

[29] Montoya M.F. Diagnostic Outcomes of Digital Images for Comprehensive Examination in Pediatric Dentistry: An Intraexaminer Agreement Assessment. https://digitalcommons.library.uab.edu/cgi/viewcontent.cgi?article=1066&context=etd-collection.

[30] Groman R., Ginsburg J. (2004). Racial and ethnic disparities in health care: A position paper of the American College of Physicians. Ann. Intern. Med..

[31] Freire-Maia J., Clementino L.C., Martins-Júnior P.A., Freire-Maia F.B. (2021). Interest in oral health education through digital technologies: A cross-sectional study. Gen. Dent..

[32] Edwards J., Waite-Jones J., Schwarz T., Swallow V. (2021). Digital Technologies for Children and Parents Sharing Self-Management in Childhood Chronic or Long-Term Conditions: A Scoping Review. Children.

[33] Yuzbasioglu E., Kurt H., Turunc R., Bilir H. (2014). Comparison of digital and conventional impression techniques: Evaluation of patients’ perception, treatment comfort, effectiveness and clinical outcomes. BMC Oral Health.

[34] Pisano M., Bramanti A., Menditti D., Sangiovanni G., Santoro R., Amato A. (2023). Modern Approaches to Providing Telematics Oral Health Services in Pediatric Dentistry: A Narrative Review. Appl. Sci..

